# Lyme Carditis Beyond Atrioventricular Block: Cardiac Arrest and Transient Atrial Fibrillation With Preserved Atrioventricular Conduction

**DOI:** 10.1155/cric/6360084

**Published:** 2026-10-06

**Authors:** Jamal A. Anthony, Katherine Doktor

**Affiliations:** ^1^ Department of Infectious Diseases Cooper University Hospital Camden New Jersey USA cooperhealth.org; ^2^ Cooper Medical School of Rowan University Camden New Jersey USA rowan.edu

## Abstract

Lyme disease may involve the heart during early dissemination and most commonly produces atrioventricular (AV) conduction abnormalities. PR interval prolongation, therefore, frequently guides clinical suspicion and management. We describe a previously healthy man who presented with seizure‐like activity, aborted cardiac arrest, transient atrial fibrillation, and cardiac magnetic resonance findings consistent with acute myocarditis, although AV conduction remained normal. Serologic testing subsequently confirmed Lyme carditis. The patient recovered completely with oral doxycycline. This case illustrates that electrical instability may occur despite preserved PR intervals and reinforces the need for suspicion in patients with compatible systemic features.

## 1. Introduction

Lyme carditis is an early disseminated manifestation of *Borrelia* infection and most often involves the conduction system. AV block, which may fluctuate rapidly, frequently necessitates hospitalization and guides the route of antimicrobial therapy [1–3]. However, myocardial involvement is heterogeneous. Pathologic and imaging studies demonstrate that inflammatory infiltration may extend beyond nodal tissue into nonconduction myocardium [4]. When this occurs, ventricular dysfunction and tachyarrhythmias may predominate despite preserved conduction [5]. Such presentations remain less emphasized and may delay recognition. We report a patient with aborted cardiac arrest and imaging‐confirmed myocarditis in whom AV conduction remained intact.

## 2. Case Presentation

A previously healthy male in his early 30s presented after an episode of witnessed seizure‐like activity for 5 min, followed by unresponsiveness and cardiac arrest at home. The patient′s wife saw the event, performed cardiopulmonary resuscitation, and achieved return of spontaneous circulation at home. Emergency medical services found the patient unresponsive with a pulse but hypotensive and bradycardic to the 40s on arrival at his residence. The bradycardia appeared to be transient, and atropine, epinephrine, or any chronotropic drugs were not administered. However, hypotension was sustained; therefore, two boluses of intravenous phenylephrine were administered, to which the patient responded. On presentation to the emergency room, he was intubated for airway protection and started on vasopressors. An electrocardiogram on presentation showed atrial fibrillation with rapid ventricular response (Figure 1), which quickly converted to normal sinus rhythm after receiving metoprolol tartrate x3 in the emergency room (Figure 2). The patient had elevated high‐sensitivity troponin levels, peaking at 2983 ng/L (NR: 0–19 ng/L).

**Figure 1:**
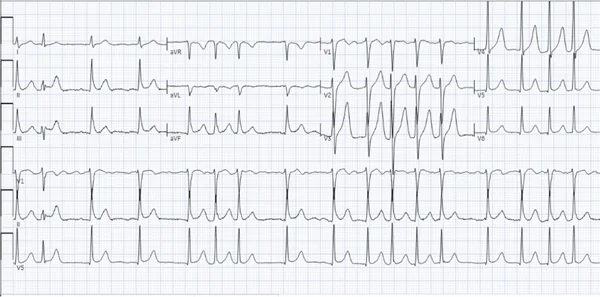
An irregular tracing with no discrete P‐waves consistent with atrial fibrillation.

**Figure 2:**
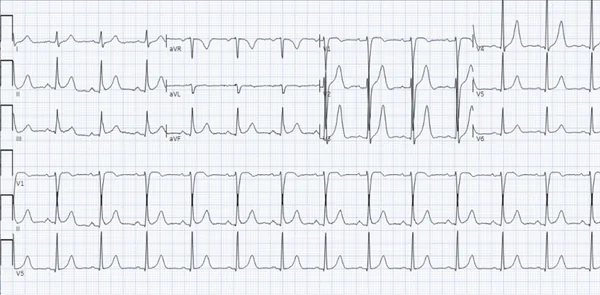
A repeat EKG after spontaneous conversion to sinus rhythm.

His echocardiogram showed basal‐to‐mid‐inferoseptal hypokinesis and a mildly depressed left ventricular function with an ejection fraction of 40%, raising concern for an ischemic etiology as the cause of his cardiac arrest. He was stabilized and transferred to the coronary care unit (CCU). There, an electroencephalogram did not demonstrate seizure activity, and it was postulated that his seizure activity was likely secondary to decreased cerebral perfusion from his cardiac arrest rather than the cause of his cardiac arrest itself. He had a cardiac catheterization, which showed no coronary artery disease (Figure 3).

**Figure 3:**
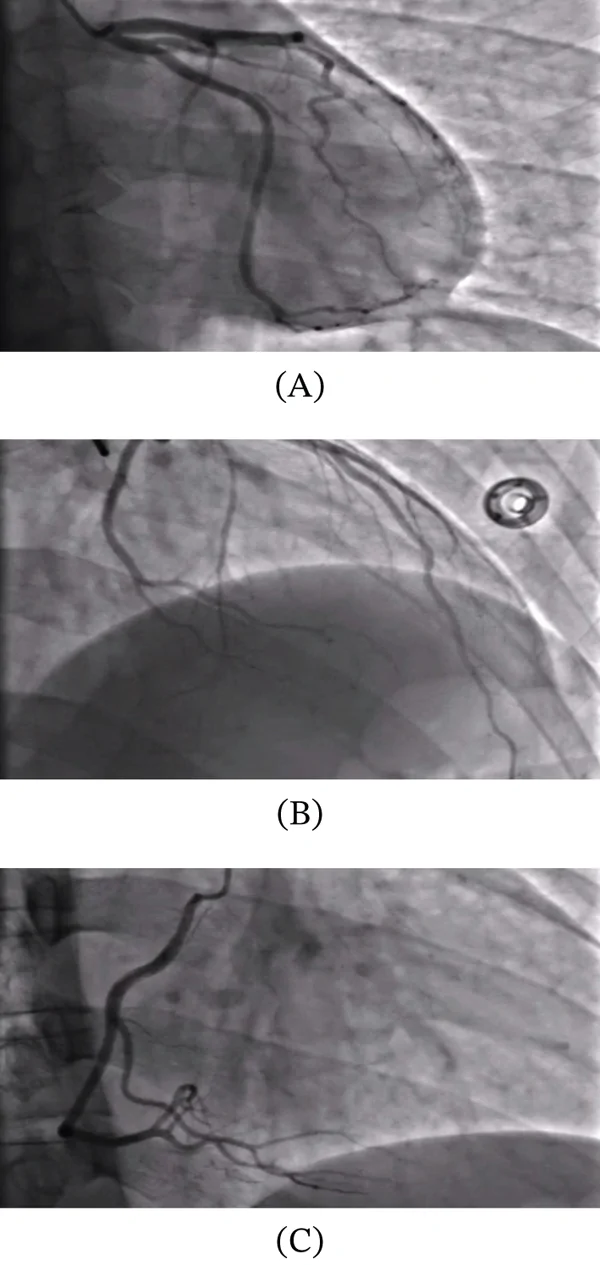
Coronary angiography without obstructive coronary artery disease. (A) Left coronary angiography obtained in a left anterior oblique caudal projection demonstrates a widely patent left main coronary artery bifurcating into the left anterior descending and left circumflex arteries without significant stenosis. (B) Right anterior oblique cranial projection of the left coronary system demonstrates a normal‐caliber left anterior descending artery and its diagonal branches with preserved distal flow and no angiographically significant obstruction. (C) Right coronary angiography in a right anterior oblique projection demonstrates a patent dominant right coronary artery with intact opacification of the posterior descending and posterolateral branches. There is no angiographic evidence of obstructive epicardial coronary artery disease.

The patient was extubated and weaned from vasopressors by Day 2 of admission and was able to provide further details. He stated that he was in his usual state of health until 1 week before presentation, when he developed a diffuse, red, pruritic, and “burning” rash on his left chest wall that started as a small papule and then became more diffuse (Figure 4A,B). This then started to resolve spontaneously. He also reported generalized headaches, malaise, myalgias, and arthralgias during the prior week. He denied any associated fever, chills, rigors, shortness of breath, chest pain, abdominal pain, nausea, vomiting, dysuria, diarrhea, or unexplained weight loss.

**Figure 4:**
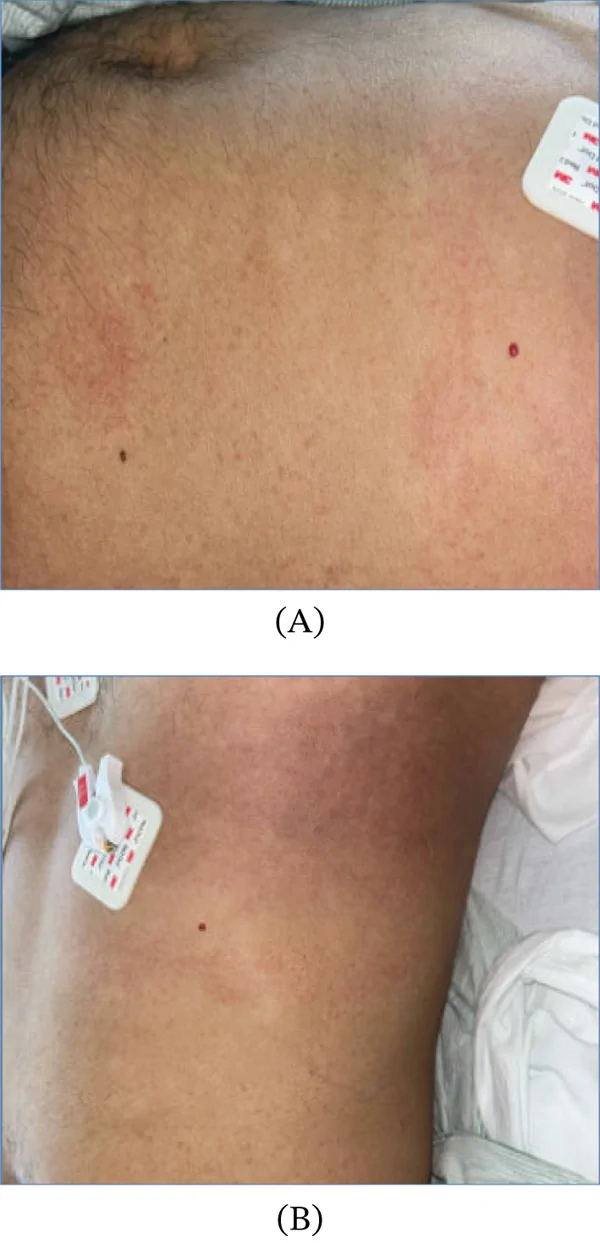
(A) A sandpaper rash on the patient′s trunk (hospital Day 4). (B) A hyperpigmented rash on the lateral chest wall (hospital Day 4).

### 2.1. Epidemiological History

He is a construction worker/carpenter who emigrated from Brazil to the United States 10 months prior to presentation. He worked as a carpenter and painted houses in Brazil. Since moving to the United States, he denied any other recent travel. He denied living in a wooded area, any insect bites, and having any pets. He denied any history of smoking or illicit drug use but occasionally drank alcohol. He denied any recent immunizations, new medications, or sick contacts. He denied knowing anyone with tuberculosis and has no family members with early‐onset heart failure. He did not endorse partaking in hiking, boating, or fishing.

### 2.2. Physical Examination

The patient was a healthy‐appearing young male who was found to be in no painful or cardiopulmonary distress. He was anicteric and acyanotic with pink and moist mucous membranes. Blood pressure was 110/58, pulse was 57 beats per minute, temperature was 97.8°F (36.6°C) (oral), respiratory rate was 15 breaths per minute, and SpO2 97% on room air. Examination was remarkable for shotty cervical lymphadenopathy; mild, nontender hepatomegaly; a faint, diffuse, sandpaper‐like rash on the trunk and back (Figure 4A); and an ovoid, purplish, hyperpigmented‐appearing rash on the left anterolateral chest wall measuring 20 × 10 cm (Figure 4B). The remainder of the examination, including cardiovascular examination, was unremarkable.

### 2.3. Differentials and Investigations

The patient had a mildly elevated WBC at presentation of 11.52 × 10^3^/uL (NR: 4.50–11.0 × 10^3^/uL), which normalized and remained within reference range throughout admission. He was noted to have a mild normocytic anemia of 11.3–13.8 g/dL (NR: 14–18 g/dL), and his platelet count was normal. C‐reactive protein was elevated at 3.78 mg/dL (NR < 0.50 mg/dL) in addition to a mild elevation of ESR at 19 mm/h (NR 0–15 mm/h). Renal and liver function were unremarkable. HIV and RPR were both nonreactive.

Given that this patient presented with an elevated troponin level after cardiac arrest, with regional wall motion abnormalities on echocardiogram, but with no evidence of coronary artery disease, his condition was further investigated with a cardiac MRI. This showed abnormalities of the basal lateral/inferolateral wall with lateral wall edema consistent with acute myocarditis. The patient′s atrial fibrillation never reoccurred. His repeat EKGs were unremarkable with a normal PR interval. Telemetry was also unremarkable.

In the setting of his myocarditis, rash, and presentation with cardiac arrest, there was a concern for an atypical presentation of a vector‐borne illness as the cause of his myocarditis and cardiac arrest.

Because he recently emigrated from Brazil, cardiac *Trypanosoma cruzi* infection was considered, as it is a common cause of cardiomyopathy and myocarditis in Latin America [6]. However, this was less likely given the absence of the typical biventricular dilated cardiomyopathy seen in cardiac trypanosomiasis [6]. *Trypanosoma cruzi* antibodies were also negative.

Given his unstable presentation and a more diffuse rash than typically seen in Lyme disease, *Rickettsia typhi* and Rocky Mountain spotted fever were also considered, but serology was negative.

Lyme disease testing was positive, with a Lyme index > 8, three reactive IgG bands, and three reactive IgM bands. In the setting of a cardiac MRI positive for myocarditis, a diagnosis of early disseminated Lyme disease with Lyme carditis was made [2, 5, 7], proving that though erythema migrans is sufficient to diagnose early localized Lyme disease [5], it is not always a classic bull′s‐eye rash, as demonstrated in this case.

The differential diagnosis also included viral myocarditis (such as Coxsackie, COVID‐19, parvovirus, HIV, and adenovirus) [8]. However, the antecedent rash, compatible systemic symptoms, nonischemic imaging findings, exclusion of coronary disease, and confirmatory serology supported Lyme carditis as the most coherent unifying explanation.

### 2.4. Treatment Outcome and Follow‐up

Given that the patient remained stable, with normal EKG and telemetry, and evidence of improved ventricular function on cardiac catheterization compared with the admission echocardiogram, he was discharged on a 21‐day course of doxycycline (100 mg PO BID). Current recommendations primarily reserve intravenous therapy for patients with high‐grade block or persistent instability attributable to conduction disease [9].

In the outpatient setting, he continued to do well. His doxycycline was continued to completion, and his outpatient EKG showed no recurrence of atrial fibrillation and no heart block. Patient is doing well with complete resolution of his myocarditis and no active heart failure (see Figure 4).

## 3. Discussion

Cardiac involvement occurs in fewer than 10% of patients with Lyme disease in the United States [5]. Atrioventricular block is traditionally emphasized as the defining feature of Lyme carditis, largely because spirochetal infiltration frequently involves the AV node and adjacent conduction tissue [1–3]. However, pathologic and cardiac magnetic resonance data demonstrate that myocardial inflammation is often multifocal and may prominently involve extranodal myocardium [4].

When inflammation is distributed in this manner, arrhythmogenesis may result from myocardial irritability, edema, and regional conduction heterogeneity rather than progressive nodal failure [5, 7].

This mechanism provides a biologically plausible framework for the electrical instability observed in our patient. He experienced cardiac arrest followed by transient post‐ROSC bradycardia and subsequently developed atrial fibrillation with rapid ventricular response, yet serial electrocardiograms demonstrated stable PR intervals without evidence of evolving AV block. Because the rhythm during cardiac arrest was not captured, the mechanism of arrest cannot be definitively established. We acknowledge that a primary bradyarrhythmia or transient ventricular tachyarrhythmia that terminated during resuscitation cannot be excluded. Importantly, however, the severity of his cardiac presentation occurred in the absence of documented AV conduction disease, supporting the possibility that the predominant inflammatory substrate involved extranodal myocardium.

Additionally, accumulating reports describe supraventricular and ventricular tachyarrhythmias in Lyme carditis that never manifest high‐grade block [5, 7, 10]. Severe and occasionally fatal outcomes have been documented, particularly when diagnosis is delayed [11]. These observations indicate a clinical phenotype that is pathophysiologically distinct from typical conduction‐dominant presentations and suggest that electrical instability may reflect myocardial inflammation capable of provoking triggered activity or reentry, independent of nodal involvement (Table 1). Although the culprit rhythm responsible for cardiac arrest in our patient remains unknown, his documented transient atrial fibrillation, MRI‐confirmed myocarditis, and preserved AV conduction are consistent with this broader spectrum of cardiac involvement.

**Table 1:** Comparison of conduction‐dominant and myocardial‐dominant presentations of Lyme carditis.

Domain	Typical: conduction‐dominant presentation	Atypical: myocardial‐dominant presentation (present case)
Objective testing	Troponin elevation; abnormal cardiac MRI; positive Lyme serology	Troponin elevation; abnormal cardiac MRI; positive Lyme serology
Primary site of involvement	Atrioventricular conduction system	Nonconduction myocardium
PR interval	Prolonged	Within normal limits
Predominant rhythm disturbance	Bradyarrhythmia/AV block	Variable: Normal sinus rhythm; tachyarrhythmia (transient or persistent); sudden unexplained cardiac arrest
Initial clinical impression	Electrical instability anticipated	May falsely appear lower risk when AV conduction is preserved
Potential clinical trajectory	Progressive conduction delay	Acute hemodynamic deterioration
Severity	Stratified by degree of AV block	May require closer monitoring; conduction severity may underestimate myocardial risk
Diagnostic challenge	Recognition driven by PR change	Requires suspicion despite normal conduction in patient with positive Lyme serology, elevated troponins, and positive MRI for myocarditis.

For the clinician at the bedside, diagnosis requires integration of clinical findings. In this case, the combination of recent rash, systemic symptoms, nonischemic late gadolinium enhancement, and angiographically normal coronary arteries created a pattern more compatible with inflammatory or infectious myocarditis than with primary ischemic disease. Structured diagnostic approaches emphasize precisely this integration of extracardiac and cardiac data when Lyme carditis is a possibility, particularly in the appropriate epidemiological context (the appropriate season in an endemic area) [12].

Current public health frameworks, including those from the Centers for Disease Control and Prevention, primarily stratify severity according to the degree of atrioventricular conduction disturbance (mild: first‐degree AV block with PR interval < 300 milliseconds; moderate: between mild and severe; severe: symptomatic, first‐degree AV block with PR interval ≥ 300 milliseconds, or second‐ or third‐degree AV block) [9]. Within such schemes, patients without PR prolongation are typically considered lower risk. Our patient, however, experienced cardiac arrest and subsequent hemodynamic instability despite the absence of documented PR prolongation or AV block, illustrating that conduction‐based classifications may not fully encompass the spectrum of myocardial involvement and disease severity. Rather than supplanting existing criteria, this case suggests that atypical presentations dominated by extranodal myocardial irritability may warrant closer monitoring and individualized clinical management.

A limitation of this case is the absence of rhythm documentation during the index cardiac arrest. Consequently, whether the arrest resulted from bradyarrhythmia, ventricular tachyarrhythmia, or another mechanism cannot be determined, and the subsequently documented atrial fibrillation should not be interpreted as the culprit rhythm.

The favorable recovery observed after antimicrobial therapy is also consistent with prior experience. Myocardial dysfunction in Lyme carditis is frequently reversible once inflammation subsides, with improvement in both rhythm stability and ventricular performance [2, 7].

The practical implication is therefore nuanced. PR interval prolongation remains an important indicator of conduction system involvement. However, when extracardiac features and imaging findings support inflammatory myocardial disease, preserved AV conduction should not preclude evaluation for Lyme carditis.

## 4. Teaching Points

• Tachyarrhythmias are increasingly recognized manifestations of Lyme carditis and may occur even when atrioventricular conduction is preserved.
• The absence of bradycardia or PR prolongation should not substantially reduce suspicion for Lyme disease in patients with compatible clinical or epidemiologic features.
• Conduction‐based severity frameworks may not fully capture presentations dominated by myocardial inflammation; such patients may benefit from closer monitoring and individualized management, as illustrated in this case.

## Funding

No funding was received for this manuscript.

## Consent

All the patients allowed personal data processing and informed consent was obtained from all individual participants included in the study.

## Conflicts of Interest

The authors declare no conflicts of interest.

## Data Availability

The data that support the findings of this study are available from the corresponding author upon reasonable request.
